# The genome sequence of the green-underside blue, *Glaucopsyche alexis* (Poda, 1761)

**DOI:** 10.12688/wellcomeopenres.17264.1

**Published:** 2021-10-15

**Authors:** Joan Carles Hinojosa Galisteo, Roger Vila, Darwin Tree of Life Barcoding collective, Wellcome Sanger Institute Tree of Life programme, Wellcome Sanger Institute Scientific Operations: DNA Pipelines collective, Tree of Life Core Informatics collective, Darwin Tree of Life Consortium

**Affiliations:** 1 Institut de Biologia Evolutiva (CSIC-UPF), Barcelona, Spain

**Keywords:** Glaucopsyche alexis, the green-underside blue, genome sequence, chromosomal

## Abstract

We present a genome assembly from an individual male *Glaucopsyche alexis* (the green-underside blue; Arthropoda; Insecta; Lepidoptera; Lycaenidae). The genome sequence is 620 megabases in span. The majority (99.87%) of the assembly is scaffolded into 23 chromosomal pseudomolecules, with the Z sex chromosome assembled.

## Species taxonomy

Eukaryota; Metazoa; Ecdysozoa; Arthropoda; Hexapoda; Insecta; Pterygota; Neoptera; Endopterygota; Lepidoptera; Glossata; Ditrysia; Papilionoidea; Lycaenidae; Polyommatinae; Glaucopsyche; *Glaucopsyche alexis* (Poda, 1761) (NCBI:txid203781).

## Introduction

*Glaucopsyche alexis* is a species of the Polyommatinae subfamily (also known as the blues) found in temperate habitats from Northwestern Africa and Western Europe to Central Asia and Amur, including the Middle East and some Mediterranean islands. However, it is absent from several major islands, including the Balearic Islands, Sardinia, Crete, Cyprus and the Atlantic archipelago of Britain and Ireland ( although a single specimen was recorded in Torquay, Devon, in September 1936). As with other Polyommatinae, adults exhibit a strong sexual dimorphism regarding the colour of the wing dorsal side: in males it is blue while, in females, it is predominantly brown. It is a univoltine species that overwinters as pupa. Adults fly during spring in most of its range, but they can fly until the beginning of summer in the coldest areas ( Tshikolovets & Others, 2011). Caterpillars feed on a wide variety of Fabaceae; they are facultative myrmecophilous and tended by various ant taxa from the subfamilies Myrmicinae and Formicinae ( Álvarez *et al.,* 2012; Tolman & Lewington, 2008). This species has an overall stable population trend and it is listed as Least Concern in the IUCN Red List ( van Swaay *et al.*, 2013).

## Genome sequence report

**Figure 1.**
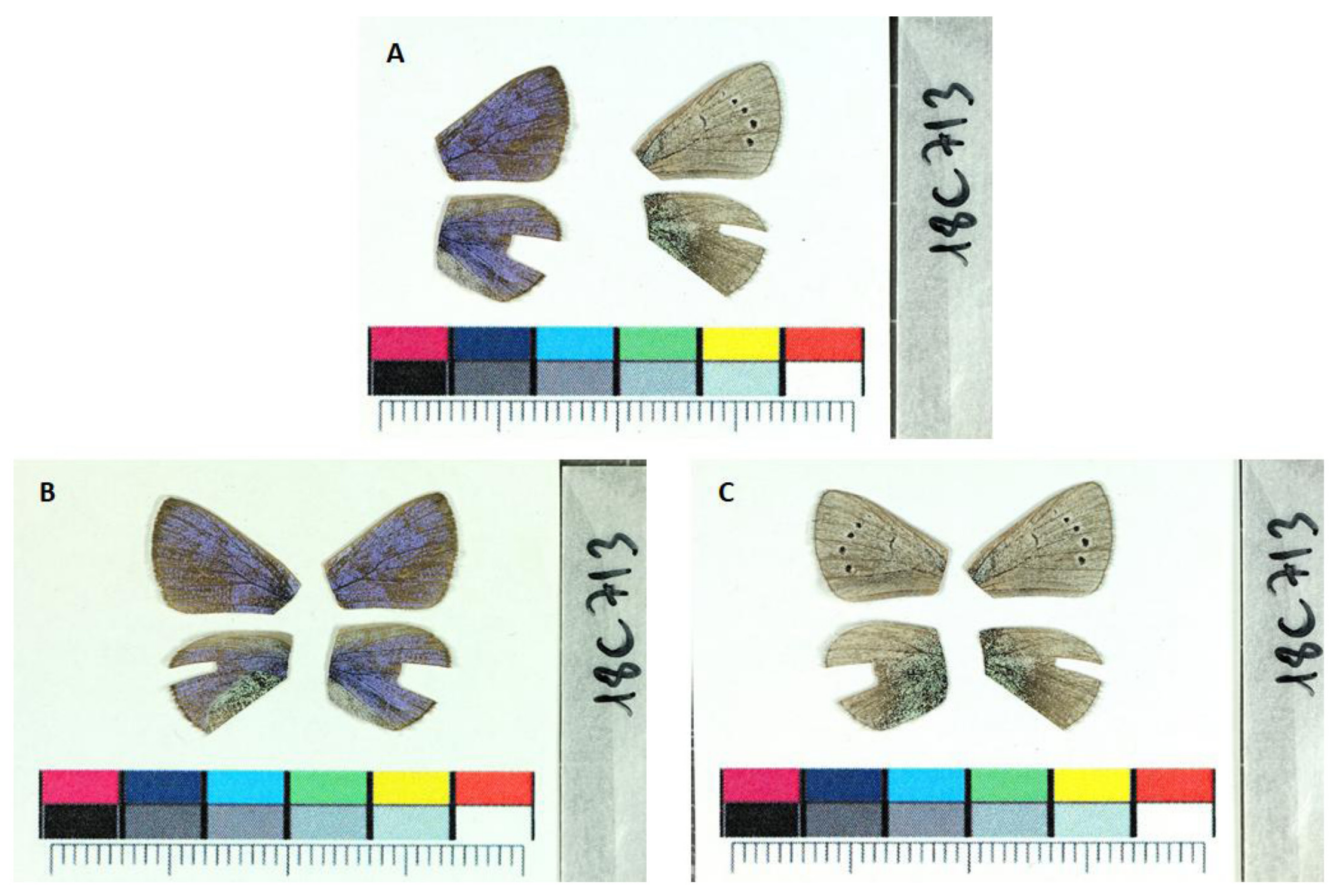
Fore and hind wings of the *Glaucopsyche alexis* specimen from which the genome was sequenced. ( **A**) Dorsal (left) and ventral (right) surface view of wings from specimen ilGlaAlex1 from Alcalá de la Selva, Teruel, Aragon, Spain, used to generate Pacific Biosciences and 10X genomics data. ( **B**) Dorsal surface view of wings from specimen ilGlaAelx1. ( **C**) Ventral surface view of wings from specimen ilGlaAelx1.

The male *G. alexis* specimen ( Figure 1) was collected from Alcalá de la Selva, Teruel, Aragon, Spain (latitude 40.3638, longitude -0.7269). The genome was sequenced from a single male *G. alexis* to 43-fold coverage in Pacific Biosciences single-molecule long reads and 75-fold coverage in 10X Genomics read clouds. Primary assembly contigs were scaffolded with chromosome conformation Hi-C data. Manual assembly curation corrected 120 missing/misjoins and removed 21 haplotypic duplications, reducing the assembly size by 0.75% and scaffold number by 5.31%, and increasing the scaffold N50 by 7.99%.

**Figure 2.**
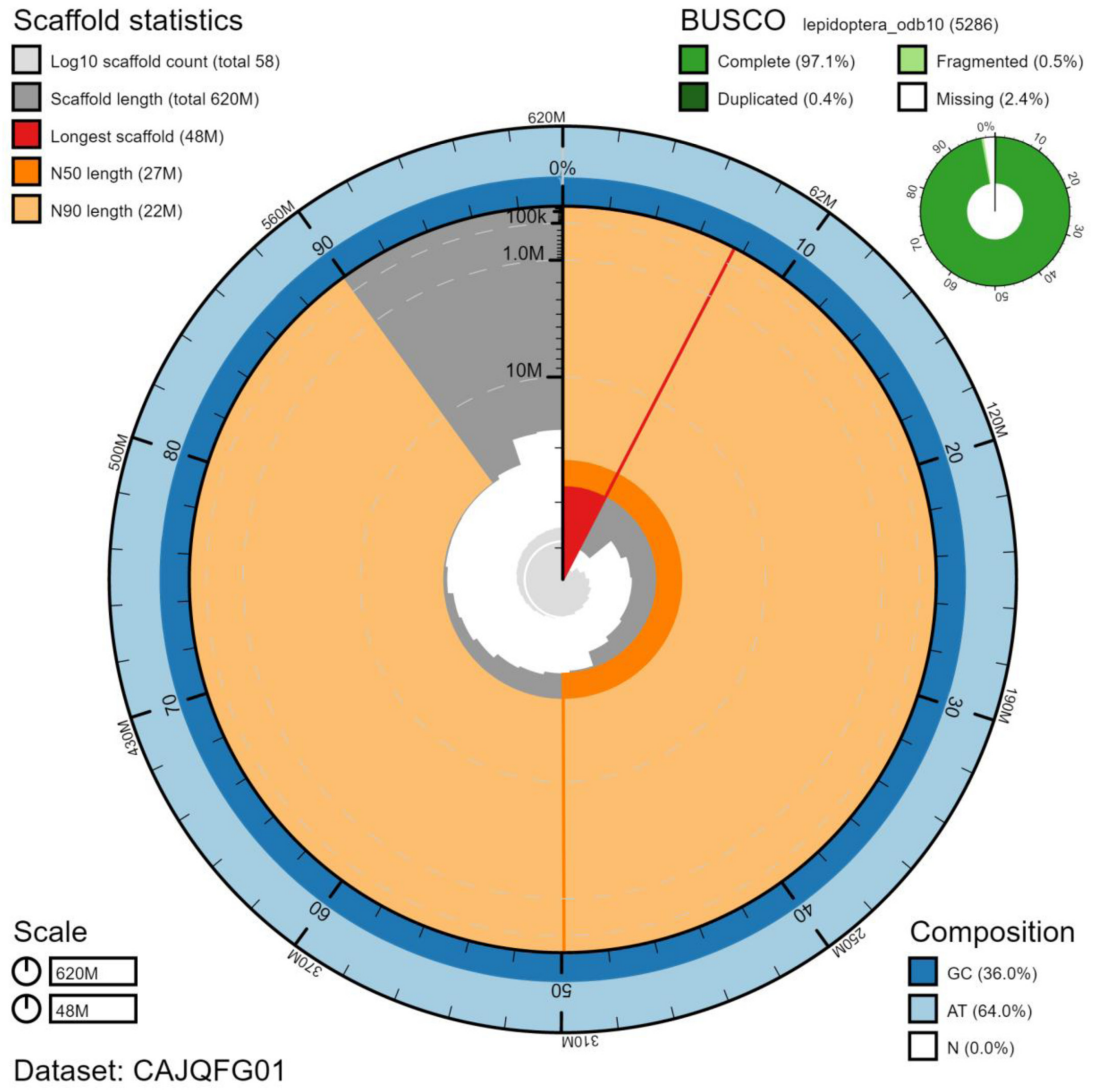
Genome assembly of *Glaucopsyche alexis*, ilGlaAlex1.1: metrics. The BlobToolKit Snailplot shows N50 metrics and BUSCO gene completeness. The main plot is divided into 1,000 size-ordered bins around the circumference with each bin representing 0.1% of the 619,543,730 bp assembly. The distribution of scaffold lengths is shown in dark grey with the plot radius scaled to the longest chromosome present in the assembly (47,686,528 bp, shown in red). Orange and pale-orange arcs show the N50 and N90 chromosome lengths (26,518,193 and 22,044,104 bp), respectively. The pale grey spiral shows the cumulative scaffold count on a log scale with white scale lines showing successive orders of magnitude. The blue and pale-blue area around the outside of the plot shows the distribution of GC, AT and N percentages in the same bins as the inner plot. A summary of complete, fragmented, duplicated and missing BUSCO genes in the lepidoptera_odb10 set is shown in the top right. An interactive version of this figure is available at https://blobtoolkit.genomehubs.org/view/ilGlaAlex1.1/dataset/CAJQFG01/snail.

The final assembly has a total length of 620 Mb in 58 sequence scaffolds with a scaffold N50 of 27 Mb ( Table 1). Of the assembly sequence, 99.87% was assigned to 23 chromosomal-level scaffolds, representing 22 autosomes (numbered by sequence length), and the Z sex chromosome ( Figure 2– Figure 5; Table 2). The assembly has a BUSCO v5.1.2 ( Simão *et al.*, 2015) completeness of 97.1% (single 96.7%; duplicated 0.4%; fragmented 0.5%; missing 2.4%) using the lepidoptera_odb10 reference set ( Table 1). While not fully phased, the assembly deposited is of one haplotype. Contigs corresponding to the second haplotype have also been deposited. The mitochondrial genome was also assembled, with a total length of 15.2 kb.

**Table 1.** Genome data for *Glaucopsyche alexis*, ilGlaAlex1.1.

*Project accession data*	
Assembly identifier	ilGlaAlex1.1
Species	*Glaucopsyche alexis*
Specimen	ilGlaAlex1
NCBI taxonomy ID	NCBI:txid203781
BioProject	PRJEB43798
BioSample ID	SAMEA7524616
Isolate information	Male, whole organism
*Raw data accessions*	
PacificBiosciences SEQUEL II	ERR6436366
10X Genomics Illumina	ERR6054614-ERR6054617
Hi-C Illumina	ERR6054618
*Genome assembly*	
Assembly accession	GCA_905404095.1
*Accession of alternate haplotype*	GCA_905404225.1
Span (Mb)	620
Number of contigs	207
Contig N50 length (Mb)	8
Number of scaffolds	58
Scaffold N50 length (Mb)	27
Longest scaffold (Mb)	39
BUSCO * genome score	C:97.1%[S:96.7%,D:0.4%], F:0.5%,M:2.4%,n:5286

*BUSCO scores based on the lepidoptera_odb10 BUSCO set using v5.1.2. C= complete [S= single copy, D=duplicated], F=fragmented, M=missing, n=number of orthologues in comparison. A full set of BUSCO scores is available at https://blobtoolkit.genomehubs.org/view/ilNymPoly1.1/dataset/CAJNAJ01/busco.

**Figure 3.**
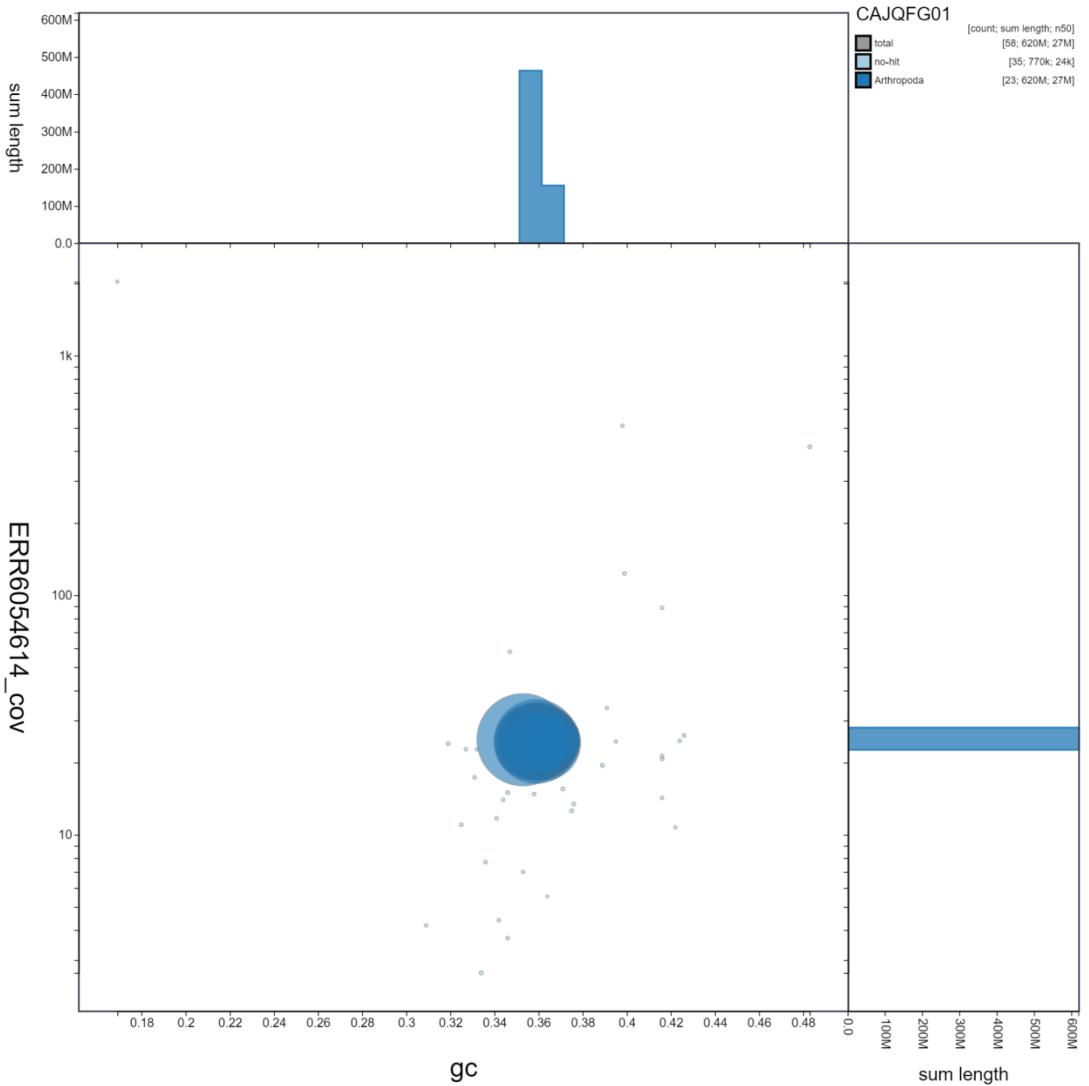
Genome assembly of *Glaucopsyche alexis*, ilGlaAlex1.1: GC coverage. BlobToolKit GC-coverage plot. Scaffolds are coloured by phylum. Circles are sized in proportion to scaffold length. Histograms show the distribution of scaffold length sum along each axis. An interactive version of this figure is available at https://blobtoolkit.genomehubs.org/view/ilGlaAlex1.1/dataset/CAJQFG01/blob.

**Figure 4.**
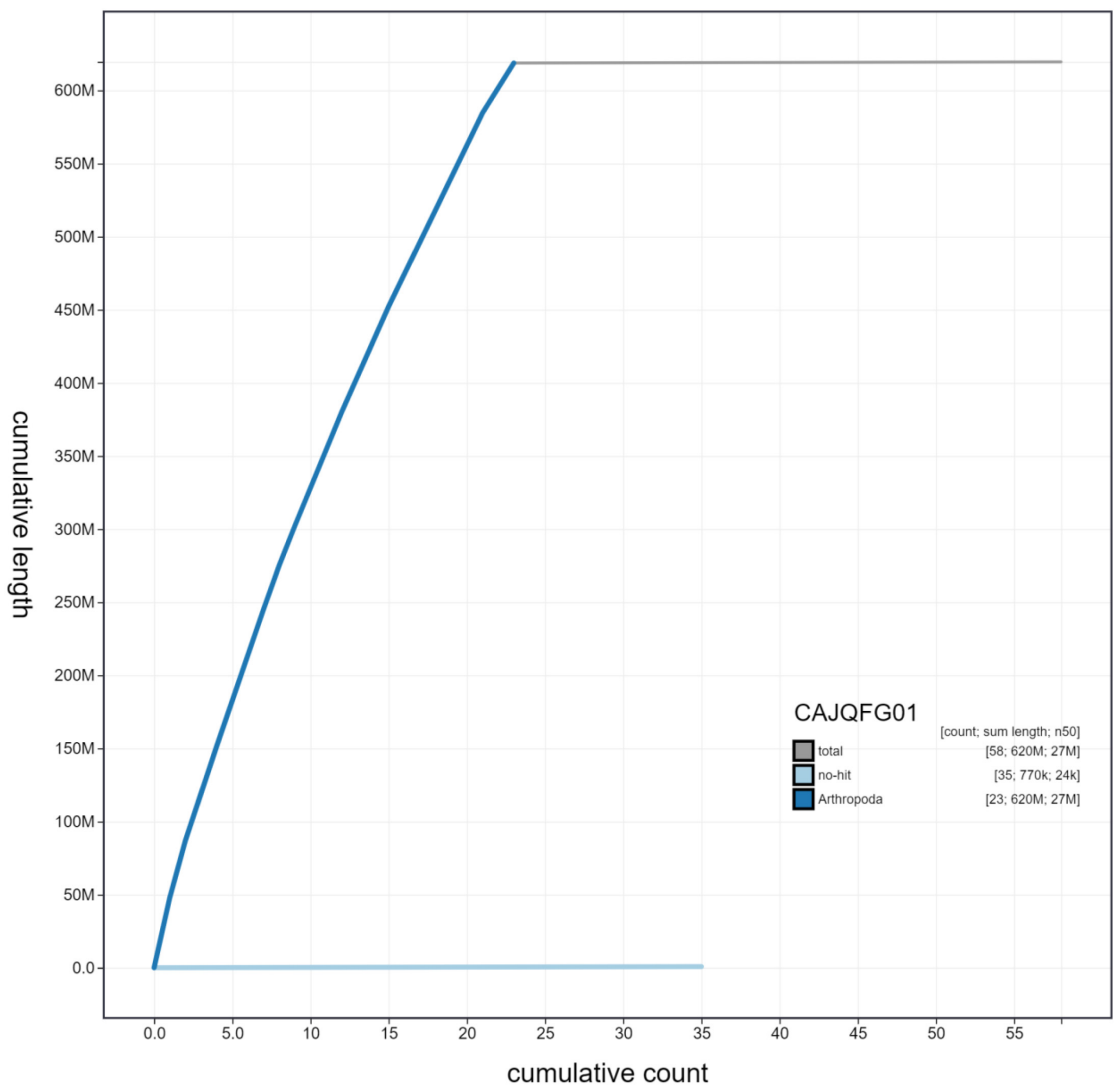
Genome assembly of *Glaucopsyche alexis*, ilGlaAlex1.1: cumulative sequence. BlobToolKit cumulative sequence plot. The grey line shows cumulative length for all scaffolds. Coloured lines show cumulative lengths of scaffolds assigned to each phylum using the buscogenes taxrule. An interactive version of this figure is available at https://blobtoolkit.genomehubs.org/view/ilGlaAlex1.1/dataset/CAJQFG01/cumulative.

**Figure 5.**
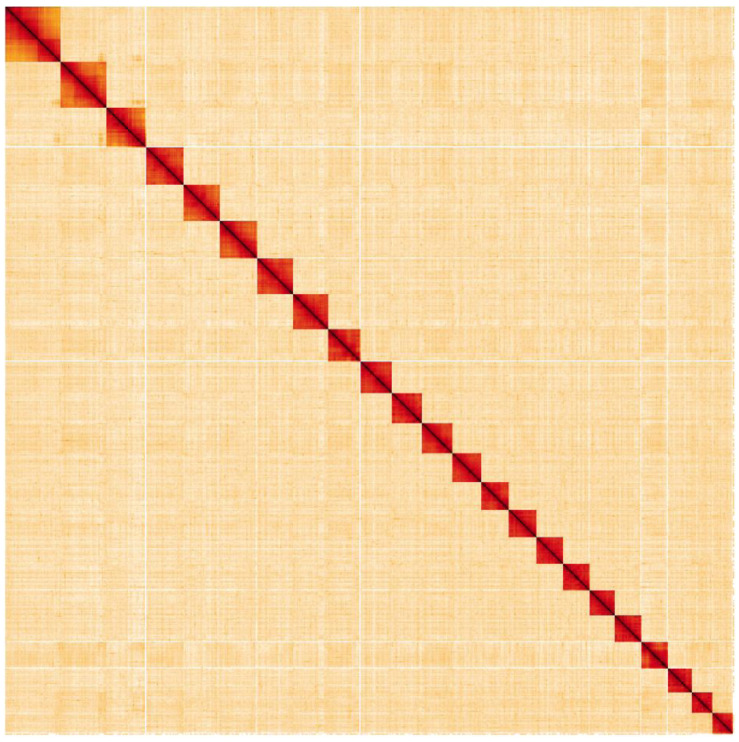
Genome assembly of *Glaucopsyche alexis*, ilGlaAlex1.1: Hi-C contact map. Hi-C contact map of the ilGlaAlex1.1 assembly, visualised in HiGlass. Chromosomal scaffolds are organised in size order from left to right and top to bottom.

**Table 2.** Chromosomal pseudomolecules in the genome assembly of *Glaucopsyche alexis*, ilGlaAlex1.1.

INSDC accession	Chromosome	Size (Mb)	GC%
FR990043.1	1	39.17	35.9
FR990044.1	2	33.01	36
FR990045.1	3	31.81	35.9
FR990046.1	4	31.46	36.2
FR990047.1	5	31.38	35.7
FR990048.1	6	30.88	35.8
FR990049.1	7	29.90	36
FR990050.1	8	26.97	36.1
FR990051.1	9	26.52	36.0
FR990052.1	10	25.96	35.9
FR990053.1	11	25.55	36.1
FR990054.1	12	24.58	36.1
FR990055.1	13	23.88	36.1
FR990056.1	14	23.50	36.2
FR990057.1	15	22.56	36.4
FR990058.1	16	22.17	36.0
FR990059.1	17	22.13	35.9
FR990060.1	18	22.05	35.9
FR990061.1	19	22.04	36.4
FR990062.1	20	21.40	36.3
FR990063.1	21	17.19	36.6
FR990064.1	22	16.97	36.4
FR990042.1	Z	47.69	35.3
FR990065.1	MT	0.02	17.3
-	Unplaced	0.75	37.1

## Methods

The male *G. alexis* specimen was collected on 12 June 2019 using a net from Alcalá de la Selva, Teruel, Aragon, Spain (latitude 40.3638, longitude -0.7269) by Joan Carles Hinojosa (Institut de Biologia Evolutiva, Barcelona), and identified by Joan carles Hinojosa and Roger Vila (Institut de Biologia Evolutiva, Barcelona). The specimen was snap-frozen from live in liquid nitrogen.

DNA was extracted at the Tree of Life laboratory, WSI. The ilGlaAlex1 sample was weighed and dissected on dry ice with tissue set aside for Hi-C sequencing. Tissue from the whole organism was disrupted to a fine powder using a powermasher. Fragment size analysis of 0.01-0.5 ng of DNA was then performed using an Agilent FemtoPulse. High molecular weight (HMW) DNA was extracted using the Qiagen MagAttract HMW DNA extraction kit. Low molecular weight DNA was removed from a 200-ng aliquot of extracted DNA using 0.8X AMpure XP purification kit prior to 10X Chromium sequencing; a minimum of 50 ng DNA was submitted for 10X sequencing. HMW DNA was sheared into an average fragment size between 12-20 kb in a Megaruptor 3 system with speed setting 30. Sheared DNA was purified by solid-phase reversible immobilisation using AMPure PB beads with a 1.8X ratio of beads to sample to remove the shorter fragments and concentrate the DNA sample. The concentration of the sheared and purified DNA was assessed using a Nanodrop spectrophotometer and Qubit Fluorometer and Qubit dsDNA High Sensitivity Assay kit. Fragment size distribution was evaluated by running the sample on the FemtoPulse system.

Pacific Biosciences HiFi circular consensus and 10X Genomics read cloud DNA sequencing libraries were constructed according to the manufacturers’ instructions. DNA sequencing was performed by the Scientific Operations core at the WSI on Pacific Biosciences SEQUEL II and Illumina HiSeq X instruments. Hi-C data were generated from abdomen tissue using the Arima v2.0 kit and sequenced on HiSeq X.

Assembly was carried out with HiCanu ( Nurk *et al.*, 2020); haplotypic duplication was identified and removed with purge_dups ( Guan *et al.*, 2020). One round of polishing was performed by aligning 10X Genomics read data to the assembly with longranger align, calling variants with freebayes ( Garrison & Marth, 2012). The assembly was then scaffolded with Hi-C data ( Rao *et al.*, 2014) using SALSA2 ( Ghurye *et al.*, 2019). The assembly was checked for contamination and corrected using the gEVAL system ( Chow *et al.*, 2016) as described previously ( Howe *et al.*, 2021). Manual curation was performed using gEVAL, HiGlass ( Kerpedjiev *et al.*, 2018) and Pretext. The mitochondrial genome was assembled using MitoHiFi ( Uliano-Silva *et al.*, 2021). The genome was analysed and BUSCO scores generated within the BlobToolKit environment ( Challis *et al.*, 2020). Table 3 contains a list of all software tool versions used, where appropriate.

**Table 3.** Software tools used.

Software tool	Version	Source
HiCanu	2.1	Nurk *et al.*, 2020
purge_dups	1.2.3	Guan *et al.*, 2020
longranger	2.2.2	https://support.10xgenomics.com/genome-exome/ software/pipelines/latest/advanced/other-pipelines
freebayes	1.3.1-17-gaa2ace8	Garrison & Marth, 2012
SALSA2	2.2	Ghurye *et al.*, 2019
MitoHiFi	1.0	Uliano-Silva *et al.*, 2021
gEVAL	N/A	Chow *et al.*, 2016
HiGlass	1.11.6	Kerpedjiev *et al.*, 2018
PretextView	0.1.x	https://github.com/wtsi-hpag/PretextView
BlobToolKit	2.6.2	Challis *et al.*, 2020

The materials that have contributed to this genome note were supplied by a Tree of Life collaborator. The Wellcome Sanger Institute employs a process whereby due diligence is carried out proportionate to the nature of the materials themselves, and the circumstances under which they have been/are to be collected and provided for use. The purpose of this is to address and mitigate any potential legal and/or ethical implications of receipt and use of the materials as part of the research project, and to ensure that in doing so we align with best practice wherever possible.

The overarching areas of consideration are:

- Ethical review of provenance and sourcing of the material;
- Legality of collection, transfer and use (national and international).

Each transfer of samples is undertaken according to a Research Collaboration Agreement or Material Transfer Agreement entered into by the Tree of Life collaborator, Genome Research Limited (operating as the Wellcome Sanger Institute) and in some circumstances other Tree of Life collaborators.

## Data availability

European Nucleotide Archive: Glaucopsyche alexis (green-underside blue). Accession number PRJEB43798; https://identifiers.org/ena.embl:PRJEB43798.

The genome sequence is released openly for reuse. The *G. alexis* genome sequencing initiative is part of the Darwin Tree of Life (DToL) project. All raw sequence data and the assembly have been deposited in INSDC databases. Raw data and assembly accession identifiers are reported in Table 1.

## References

[ref-1] Álvarez M Munguira LM Martínez-Ibáñez MD : Nuevos Datos Y Recopilación de Las Relaciones Entre Lycaenidae Y Formicidae En La Península Ibérica (Lepidoptera: Lycaenidae; Hymenoptera: Formicidae). *SHILAP Revista de Lepidopterologica.* 2012;40(157):45–59. Reference Source

[ref-2] Challis R Richards E Rajan J : BlobToolKit - Interactive Quality Assessment of Genome Assemblies. *G3 (Bethesda).* 2020;10(4):1361–74. 10.1534/g3.119.400908 32071071 PMC7144090

[ref-3] Chow W Brugger K Caccamo M : gEVAL - a web-based browser for evaluating genome assemblies. *Bioinformatics.* 2016;32(16):2508–10. 10.1093/bioinformatics/btw159 27153597 PMC4978925

[ref-4] Garrison E Marth G : Haplotype-Based Variant Detection from Short-Read Sequencing. arXiv: 1207.3907.2012. Reference Source

[ref-5] Ghurye J Rhie A Walenz BP : Integrating Hi-C Links with Assembly Graphs for Chromosome-Scale Assembly. *PLoS Comput Biol.* 2019;15(8):e1007273. 10.1371/journal.pcbi.1007273 31433799 PMC6719893

[ref-6] Guan D McCarthy SA Wood J : Identifying and Removing Haplotypic Duplication in Primary Genome Assemblies. *Bioinformatics.* 2020;36(9):2896–98. 10.1093/bioinformatics/btaa025 31971576 PMC7203741

[ref-7] Howe K Chow W Collins J : Significantly Improving the Quality of Genome Assemblies through Curation. *GigaScience.* 2021;10(1):giaa153. 10.1093/gigascience/giaa153 33420778 PMC7794651

[ref-8] Kerpedjiev P Abdennur N Lekschas F : HiGlass: Web-Based Visual Exploration and Analysis of Genome Interaction Maps. *Genome Biol.* 2018;19(1):125. 10.1186/s13059-018-1486-1 30143029 PMC6109259

[ref-9] Nurk S Walenz BP Rhie A : HiCanu: Accurate Assembly of Segmental Duplications, Satellites, and Allelic Variants from High-Fidelity Long Reads. *Genome Res.* 2020;30(9):1291–1305. 10.1101/gr.263566.120 32801147 PMC7545148

[ref-10] Rao SSP Huntley MH Durand NC : A 3D Map of the Human Genome at Kilobase Resolution Reveals Principles of Chromatin Looping. *Cell.* 2014;159(7):1665–80. 10.1016/j.cell.2014.11.021 25497547 PMC5635824

[ref-11] Simão FA Waterhouse RM Ioannidis P : BUSCO: Assessing Genome Assembly and Annotation Completeness with Single-Copy Orthologs. *Bioinformatics.* 2015;31(19):3210–12. 10.1093/bioinformatics/btv351 26059717

[ref-13] Tolman T Lewington R : The Most Complete Guide to the Butterflies of Britain and Europe. Harper Collins, London.2008. Reference Source

[ref-14] Tshikolovets VV and Others : Butterflies of Europe & the Mediterranean Area. *Butterflies of Europe & the Mediterranean Area.* 2011. Reference Source

[ref-15] Uliano-Silva M Nunes JGF Krasheninnikova K : marcelauliano/MitoHiFi: mitohifi_v2.0.2021. 10.5281/zenodo.5205678

[ref-12] van Swaay C Wynhoff I Verovnik R : IUCN Red List of Threatened Species: Glaucopsyche Alexis. *IUCN Red List of Threatened Species.* 2013. Reference Source

