# Supplementary material for: The genome sequence of the green-underside blue, Glaucopsyche alexis (Poda, 1761)
Source: Wellcome Open Res. 2021 Oct 15;6:274. [Version 1] doi: 10.12688/wellcomeopenres.17264.1 (PMC10349268; doi:10.12688/wellcomeopenres.17264.1)
Supplement: Peer review history

# Peer review: Reviewer response for version 1

**DOI:** 10.21956/wellcomeopenres.19082.r60757

**Published:** 2023-07-14

**Authors:** Nicola J Nadeau

**Affiliations:** 1 The University of Sheffield, Sheffield, England, UK

The article presents a chromosome-level assembly of the Lycaenid butterfly *Glaucopsyche alexis*. The assembly has been generated using accepted standard methodologies and appears of excellent quality. Perhaps the introduction could be expanded slightly to give more context to how the genome might be used (what research questions will it help to address) and perhaps how it relates to existing genome sequences (in terms of taxonomic coverage of the group). Figure 5 is not fully explained for a reader who is not familiar with what a "Hi-C contact map" is.

Are sufficient details of methods and materials provided to allow replication by others?

Yes

Is the rationale for creating the dataset(s) clearly described?

Partly

Are the datasets clearly presented in a useable and accessible format?

Partly

Are the protocols appropriate and is the work technically sound?

Yes

Reviewer Expertise:

Butterfly evolutionary genomics

I confirm that I have read this submission and believe that I have an appropriate level of expertise to confirm that it is of an acceptable scientific standard.

# Peer review: Reviewer response for version 1

**DOI:** 10.21956/wellcomeopenres.19082.r51822

**Published:** 2022-08-04

**Authors:** Victoria Twort

**Affiliations:** 1 University of Helsinki, Helsinki, Finland

The authors present the genome sequence of *Glaucopsyche alexis *following the approach used by the Darwin Tree of Life Project. **The genome is of high quality and freely available. 

Some minor suggestions:

1. With regards to the methods used, it would useful if the computational methods used included settings or perhaps a common github or container that has scripts/actual commands used. So others can install/run a similar pipeline. As it stands it is unclear how one would replicate this.
2. Was the mitochondrial genome annotated or check in any way for accuracy/completeness? It is only mentioned in passing.
3. It isn't clear at the beginning of the methods which tissue is dissected out for Hi-C sequencing, although it is mentioned later in the note. You could change the line to read 'abdomen tissue' was set aside for Hi-C. As it is currently unclear if it was only abdomen tissue that was dissected or were others removed as well?
4. From experience, it is difficult to obtain assemblies without contamination. There is no mention here about the presence of sequences from other organisms, were some sort of measure taken to avoid contamination from the gut, ectoparasites etc? If there was, it would be useful for others to mention what you did to achieve this.

Are sufficient details of methods and materials provided to allow replication by others?

Yes

Is the rationale for creating the dataset(s) clearly described?

Yes

Are the datasets clearly presented in a useable and accessible format?

Yes

Are the protocols appropriate and is the work technically sound?

Yes

Reviewer Expertise:

Genomics, evolution, population genetics, museomics

I confirm that I have read this submission and believe that I have an appropriate level of expertise to confirm that it is of an acceptable scientific standard.
